# Glucose metabolism characteristics and TLR8-mediated metabolic control of CD4^+^ Treg cells in ovarian cancer cells microenvironment

**DOI:** 10.1038/s41419-020-03272-5

**Published:** 2021-01-07

**Authors:** Rui Xu, Ming Wu, Shuna Liu, Wenwen Shang, Rong Li, Juan Xu, Lei Huang, Fang Wang

**Affiliations:** 1 Department of Laboratory Medicine, the First Affiliated Hospital of Nanjing Medical University, Nanjing, 210029 China; 2 National Key Clinical Department of Laboratory Medicine, Nanjing, 210029 China

**Keywords:** Glycobiology, Tumour immunology

## Abstract

Immunotherapy is expected to become the most promising new treatment for ovarian cancer owing to its immunogenicity. However, immunosuppression in the tumor microenvironment is a major obstacle to the efficacy of tumor therapy. Studies have found different metabolism ways of regulatory T cells (Tregs) in the cancer environment may be related to the immunosuppression and Toll-like receptor 8 (TLR8) can reverse the suppression function of Tregs. But it is still unclear that if the TLR8-mediated function reversal is associated with the change of glucose metabolism of Tregs. It was found that the positive expression rates of Glut1, HIF-1α, and Ki67 in CD4^+^ Treg cells of OC were significantly higher than that in benign ovarian tumor and HC, and also significantly higher than that in CD4^+^ Teffs of OC. What’s more, compared with CD4^+^ Teff group, CD4^+^ Tregs highly expressed seven genes and three proteins related to glucose metabolism and had higher levels of glucose uptake and glycolysis. After activating TLR8 signal of CD4^+^ Tregs, the proliferation level of naive CD4^+^ T cells was higher than that of the control group. At the same time, the expression levels of eight genes and five proteins related to glucose metabolism in CD4^+^ Treg cells with TLR8 activated were decreased and levels of glucose uptake and glycolysis were also lower. Furthermore, TLR8 signaling also downregulated the mTOR pathway in CD4^+^ Tregs. CD4^+^ Tregs pretreated with 2-deoxy-d-Glucose (2-DG) and galloflavin also attenuated the inhibition of Teffs proliferation. Although CD4^+^ Tregs pretreated with 2-DG and galloflavin before activating TLR8 signal had no significant difference compared with the group only treated with inhibitors, which suggested TLR8-mediated reversal of CD4^+^ Treg cells inhibitory function in ovarian cancer cells co-cultured microenvironment had a causal relationship with glucose metabolism.

## Facts

1. Glucose metabolic activities of CD4^+^ Tregs are more active than that of CD4^+^ Teffs in peripheral blood of ovarian cancer.
2. TLR8 activation may cause CD4^+^ Tregs immune-suppressive function reversed in ovarian cancer co-culture environment.
3. TLR8 activation decrease the glucose metabolism level of CD4^+^ Tregs.
4. TLR8-mediated suppressive function reversal of CD4^+^ Tregs has a causal relationship with the change of glucose metabolism in ovarian cancer co-culture environment.

## Introduction

Ovarian cancer (OC) is a common gynecological tumor with the highest mortality^1^. Because of its hidden incidence and rapid progress, the tumor is easy to spread and lead to extensive metastasis of pelvic and abdominal organs. More than half of the patients with OC had been diagnosed as advanced and the prognosis is very poor. At present, radical surgery combined with adjuvant chemotherapy is still the basic treatment, but the therapeutic effect is not very ideal^2^. About 70% of the patients recur within two years, and the 5-year survival rate is <40%^3^. Immunotherapy is expected to become the most promising new treatment for OC owing to its immunogenicity. However, immunosuppression in the tumor microenvironment is a major obstacle to the efficacy of tumor therapy and regulatory T cells (Tregs) is an important factor in the formation of immunosuppression^4–6^.

Abnormal proliferating cells such as tumor cells could produce a large amount of lactic acid and ATP through glycolysis under aerobic or anoxic conditions. This is known as the Warburg effect^7–9^. T cells in the tumor microenvironment also tend to use this metabolic approach as “metabolic reprogramming”. In the tumor microenvironment, there is a nutritional competition between immune cells and tumor cells. Tumor cells consume lots of nutrients, inhibit the aerobic glycolysis of effector T cells (Teffs) to inhibit their function, and compensate to enhance the oxidative phosphorylation of mitochondria to promote the differentiation of Tregs^10^. At the same time, tumor local inhibitory cell subsets such as Tregs also inhibit the function of antitumor cells, which makes the development of the tumor in a negative direction^11^.

Toll-like receptor (TLR) is a kind of important pattern recognition receptor, which is responsible for identifying various pathogen-related molecular patterns and is the main bridge between innate immunity and specific immunity^12–14^. TLR can regulate the differentiation and function of Tregs directly or indirectly. Among these TLR signals, TLR8 is highly expressed on Tregs, and its ligands can reverse the immunosuppressive function of Tregs. Moreover, TLR8 signal and Foxp3 can regulate the balance of mTORC1 signal pathway and glucose metabolism, thus regulating the proliferation and immunosuppressive function of Tregs^15^.

In this study, we found more active glucose metabolism of CD4^+^ Tregs than CD4^+^ Teffs in peripheral blood of patients with OC and that of patients with benign ovarian tumor (BOT) and healthy control (HC), suggesting that it may have a better effect on tumor immunotherapy with CD4^+^ Tregs as target cells. It was further found TLR8-mediated reversal of CD4^+^ Tregs inhibitory function in OC cells co-cultured microenvironment has a causal relationship with glucose metabolism. Regulating the metabolism of CD4^+^ Tregs cells may provide new clues for the immunotherapy of OC.

## Results

### Glucose metabolism-related factors were highly expressed in CD4^+^ Tregs of OC

**Fig. 1:**
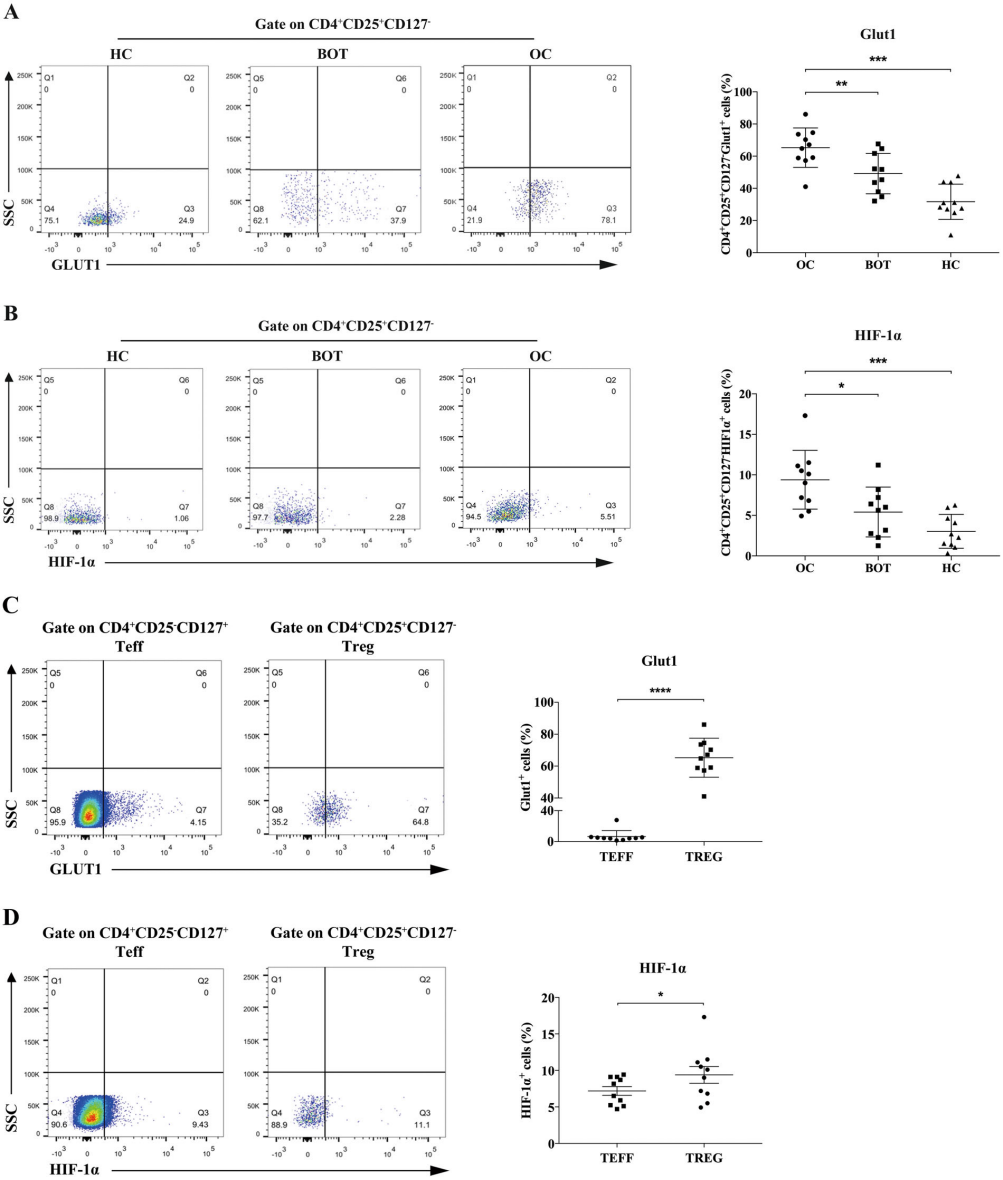
Glucose metabolism-related factors expression of CD4^+^ Tregs and Teffs in peripheral blood. **A**, **B** Expression of Glut1 and HIF-1α in CD4^+^ Tregs of human ovarian cancer (*n* = 10) peripheral blood, benign ovarian cancer (*n* = 10) peripheral blood and healthy control (*n* = 10) peripheral blood, as detected via flow cytometry. Left images are the representative flow cytometric analysis and plots are gate on CD4^+^CD25^+^CD127^−^. Right bar diagram summarizes the expression ratios as the mean ± SEM; *P < 0.05; **P < 0.01; ***P < 0.001. **C**, **D** Left images are the representative flow cytometric analysis of Glut1 and HIF-1α in CD4^+^ Tregs and Teffs of OC (*n* = 10) and plots are gate on CD4^+^CD25^+^CD127^−^ or CD4^+^CD25^−^CD127^+^. Right bar diagram shows the proportions of Glut1 and HIF-1α in CD4^+^ Tregs and Teffs. Data are displayed as mean ± SEM; *P < 0.05; ****P < 0.0001.

We used flow cytometry to investigate the expression of glucose transporter 1 (Glut1) and hypoxia inducing factor 1α (HIF-1α), which are important for glucose metabolism. Tregs were identified as CD4^+^CD25^+^CD127^−^ T cells and Teffs were CD4^+^CD25^−^CD127^+^ T cells. As shown in Fig. 1A, we found that the expression ratio of Glut1 from Tregs showed obvious difference in OC patients, BOT patients and HCs ((65.62% ± 11.93%) vs (49.17% ± 11.93%) vs (31.67% ± 10.40%); *P* < 0.01, *P* < 0.001, respectively). Besides Glut1, HIF-1α of Tregs in OC patients were also illustrated higher expression rate compared with BOT patients and HCs ((9.40% ± 3.44%) vs. (5.41% ± 2.92%) vs. (3.02% ± 1.98%); *P* < 0.05, *P* < 0.001, respectively, Fig. 1B). We also analyzed the expression differences of Glut1 and HIF-1α in Teffs between three groups and found that the differences were not as obvious as that in CD4^+^ Tregs (Fig. S1A, B). Meanwhile, we tested the Ki67^+^ subsets in CD4^+^ Tregs and the result showed higher Ki67 expression ratio of Tregs in OC patients compared with other two groups, which suggested CD4^+^ Tregs in OC patients had more active proliferation (Fig. S2A). And the high proliferation was consistent with high glycolysis metabolism. Furthermore, in OC patients, CD4^+^ Tregs had higher expression ratio of both Glut1 and HIF-1α than CD4^+^ Teffs ((65.62% ± 11.93%) vs. (6.54% ± 7.24%), *P* < 0.0001; (9.40% ± 3.44%) vs (7.20% ± 1.79%), *P* < 0.05, Fig. 1C, D). CD4^+^ Tregs also expressed more Glut1 than CD4^+^ Teffs in BOT patients and HCs, although the difference was not comparable to that of OC. Meanwhile, the expression of HIF-1α did not make difference between them (Fig. S1C-F). These results suggested that the glucose metabolism-related factors of CD4^+^ Tregs are highly expressed in peripheral blood of patients with ovarian cancer.

### CD4^+^ Treg cells had higher level of glucose metabolism in OC cells co-culture system

**Fig. 2:**
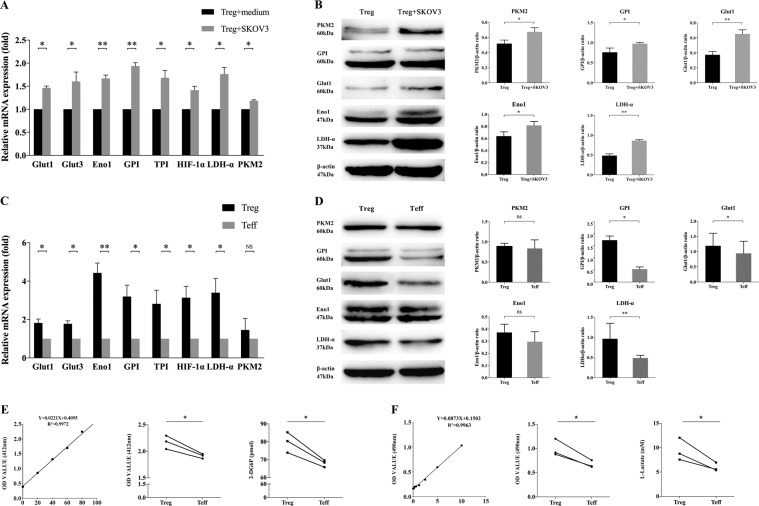
Glucose metabolism levels of CD4^+^ Tregs and Teffs co-cultured with SKOV3. **A** Expression levels of genes related to glucose metabolism (Glut1, Glut3, GPI, TPI, HIF-1α, LDH-α, PKM2, Eno1) in CD4^+^ Tregs detected via quantitative real-time PCR. Expression levels of each gene were normalized to β-actin expression level and adjusted to the levels in CD4^+^ Tregs without co-cultured with SKOV3 (served as 1). Data shown are mean ± SEM; *P < 0.05; **P < 0.01. **B** Expression levels of proteins related to glucose metabolism (Glut1, GPI, Eno1, LDH-α, PKM2) detected by western blot. The upper five panels show the western blot analysis results. The bottom panel shows the protein expressions analyzed quantitatively and compared with β-actin expression with a densitometer. Results shown in the histogram are mean ± SEM; *P < 0.05; **P < 0.01. **C** Expression levels of genes related to glucose metabolism (Glut1, Glut3, GPI, TPI, HIF-1α, LDH-α, PKM2, Eno1) in CD4^+^ Tregs and Teffs co-cultured with SKOV3 by quantitative real-time PCR. Expression levels of each gene were normalized to β-actin expression level and adjusted to the levels in CD4^+^ Teffs (served as 1). Data shown are mean ± SEM; *P < 0.05; **P < 0.01; ns *P* > 0.05. **D** Expression levels of proteins related to glucose metabolism (Glut1, GPI, Eno1, LDH-α, PKM2) detected by western blot. The upper five panels show the western blot analysis results. The bottom panel shows the protein expressions analyzed quantitatively and compared with β-actin expression with a densitometer. Results shown in the histogram are mean ± SEM; *P < 0.05; **P < 0.01; ns *P* > 0.05. **E** The levels of glucose uptake of CD4^+^ Tregs and Teffs co-cultured with SKOV3 via colourimetry. The left is standard curve and the mid shows results as OD value. The right is level of 2-DG6P by conversion. Every point represents the mean of three reduplicated wells in one experiment; *P < 0.05. **F** The levels of glycolysis of CD4^+^ Tregs and Teffs co-cultured with SKOV3 by colourimetry. The left is standard curve and the mid shows results as OD value. The right is level of l-Lactate by conversion. Every point represents the mean of three reduplicated wells in one experiment; *P < 0.05.

We next investigated the level of glucose metabolism in CD4^+^ Tregs and CD4^+^ Teffs. According to the above results, the characteristics of glucose metabolism of CD4^+^ Tregs may be obviously different in OC patients rather than HCs. Because the number of Tregs was extremely small and it was difficult to obtain blood samples from OC patients, we first built an amplification system of T cells in vitro to solve lack of cells. After amplification, the number of Tregs and Teffs could reach to (96.7 ± 9.81) times and (113.3 ± 8.16) times, respectively (Fig. S3). To simulate the OC microenvironment, we co-cultured CD4^+^ Tregs and Teffs with ovarian serous adenocarcinoma cell line SKOV3 for 3 days. As shown in Fig. 2A, B, CD4^+^ Tregs co-cultured with SKOV3 were found significantly higher levels of genes about glucose metabolism compared with normal cultured CD4^+^ Tregs. Genes tested included Glut1 and 3, as well as, HIF-1α, glucose-6-phosphate isomerase (GPI), triosephosphate isomerase (TPI), enolase 1 (ENO1), pyruvate kinase muscle 2 (PKM2), and lactate dehydrogenase A (LDH-α). At the same time, CD4^+^ Tregs co-cultured with SKOV3 also had significant increased expression of Glut1, GPI, PKM2, Eno1, and LDH-α on protein level. The level of Ki67 was also higher in CD4^+^ Tregs co-cultured with SKOV3 (Fig. S2B). These results were consistent with Fig. 1 that CD4^+^ Tregs expressed more glucose metabolism-related factors in OC patients and showed higher proliferation, which suggested that CD4^+^ Tregs co-cultured with SKOV3 system can simulate the microenvironment of OC at a certain degree. Then we found that, in co-culture microenvironment, CD4^+^ Tregs had higher expression levels of whether genes or proteins related to glucose metabolism than CD4^+^ Teffs (Fig. 2C, D). Furthermore, glucose uptake and glycolysis assays showed the same difference between CD4^+^ Tregs and CD4^+^ Teffs (Fig. 2E, F). It was pointed out CD4^+^ Tregs have more-active glucose metabolism than that of CD4^+^ Teffs in co-culture microenvironment.

### TLR8 signaling inhibits immunosuppressive function and glucose metabolism of CD4^+^ Tregs in SKOV3 co-culture microenvironment

**Fig. 3:**
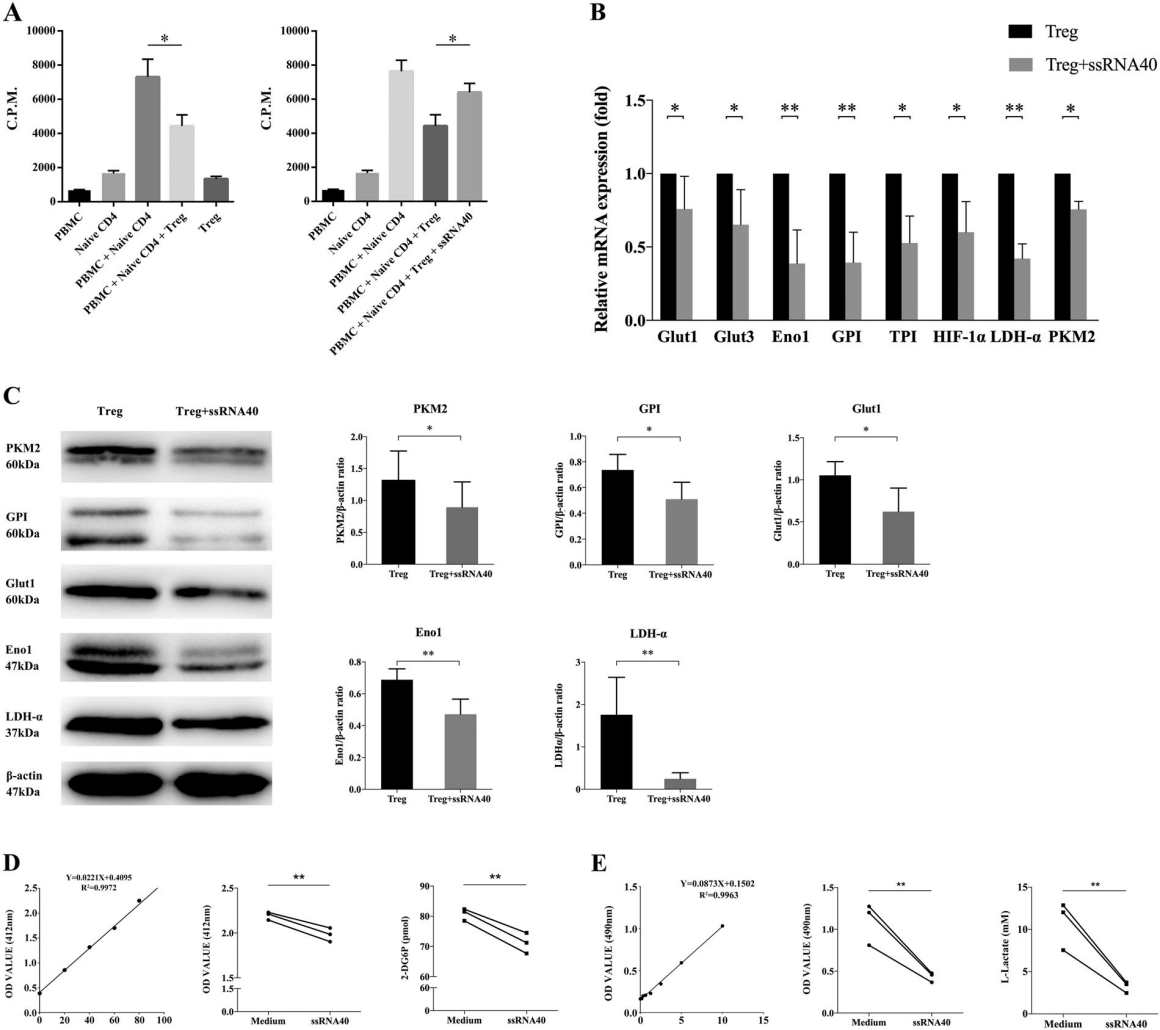
Effects of TLR8 ligands on supressive function and glucose metabolism of CD4^+^ Tregs in SKOV3 co-cultured environment. **A** After activating TLR8 signal of CD4^+^ Tregs, co-culture them with naive CD4^+^ T cells. The proliferation of co-cultured naive T cells stimulated by anti-CD3 antibody was determined by [^3^H]-thymidine incorporation assays. Data shown are mean ± SEM; *P < 0.05. **B** Expression levels of genes related to glucose metabolism (Glut1, Glut3, GPI, TPI, HIF-1α, LDH-α, PKM2, Eno1) in CD4^+^ Tregs after treated by ssRNA40 with quantitative real-time PCR. Expression levels of each gene were normalized to β-actin expression level and adjusted to the levels in CD4^+^ Tregs without TLR8 activation (served as 1). Data shown are mean ± SEM; *P < 0.05; **P < 0.01. **C** Expression levels of proteins related to glucose metabolism (Glut1, GPI, Eno1, LDH-α, PKM2) detected by western blot. The upper five panels show the western blot analysis results. The bottom panel shows the protein expressions analyzed quantitatively and compared with β-actin expression with a densitometer. Results shown in the histogram are mean ± SEM; *P < 0.05; **P < 0.01. **D** The levels of glucose uptake of CD4^+^ Tregs after treated with ssRNA40 via colorimetry. The left is standard curve and the mid shows results as OD value. The right is level of 2-DG6P by conversion. Every point represents the mean of three reduplicated wells in one experiment; *P < 0.05. **E** The levels of glycolysis of CD4^+^ Tregs after treated with ssRNA40 by colourimetry. The left is standard curve and the mid shows results as OD value. The right is level of l-Lactate by conversion. Every point represents the mean of three reduplicated wells in one experiment; *P < 0.05.

TLR can regulate the functions of T cells in direct or indirect ways. In our previous studies, we found that TLR8 ligands can reverse the inhibitory function of CD4^+^ T cells derived from OC. Meanwhile, the level of Foxp3 in CD4^+^ T cells also reduced. So, we further investigated the effects of TLR8 signaling on CD4^+^ Tregs. As shown in Fig. 3A, CD4^+^ Tregs can inhibit the proliferation of naive CD4^+^ T cells. But the proliferation level of naive CD4^+^ T cells significantly rose after activating TLR8 signaling of CD4^+^ Tregs. Then we detected glucose metabolism level of CD4^+^ Tregs after TLR8 ligand ssRNA40 treatment in SKOV3 co-culture microenvironment. Compared with cells without ssRNA40 treatment, ssRNA40 significantly decreased the expression levels of genes related to glucose metabolism (Fig. 3B). Proteins had the same results (Fig. 3C). The levels of glucose uptake and glycolysis were also down (Fig. 3D). In addition, we also activated TLR8 signal of CD4^+^ Teffs and then detected the genes and proteins related to glucose metabolism of them. As shown in Fig. S4, we found that ssRNA40 did not inhibit glucose metabolism of CD4^+^ Teffs, indicating the function of ssRNA40 had its specificity. These results suggested that activation of TLR8 signaling can reverse the immunosuppressive function of CD4^+^ Treg cells and can inhibit the glucose metabolism of CD4^+^ Tregs.

### The TLR8-mediated suppressive function reversal of CD4^+^ Tregs results from the change of glucose metabolism

**Fig. 4:**
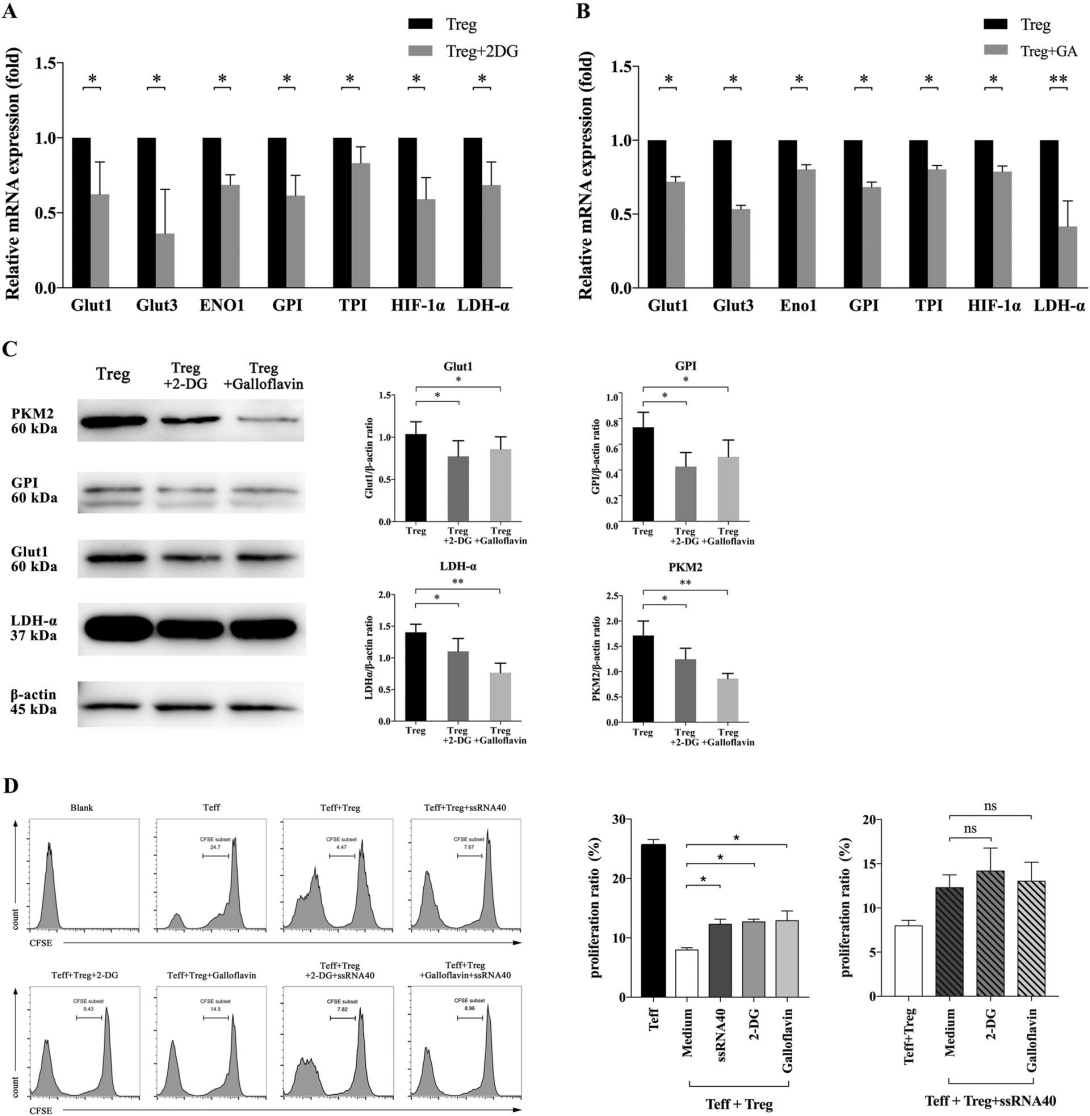
The reversal of CD4^+^ Treg suppressive function mediated by TLR8 signal may have a relationship with glucose metabolism. **A**, **B** The expression levels of glucose metabolism-related genes (Glut1, Glut3, GPI, TPI, HIF-1α, LDH-α, PKM2, Eno1) in Tregs treated with 2-DG and Galloflavin. Expression levels of each gene were normalized to β-actin expression level and adjusted to the levels in CD4^+^ Tregs without treatment (served as 1). Data shown are mean ± SEM; *P < 0.05; **P < 0.01. **C** The expression levels of glucose metabolism-related proteins (Glut1, GPI, LDH-α, PKM2) in Tregs treated with two inhibitors. The upper four panels show the western blot analysis results. The bottom panel shows the protein expressions analyzed quantitatively and compared with β-actin expression with a densitometer. Results shown in the histogram are mean ± SEM; *P < 0.05; **P < 0.01. **D** The proliferation levels of Teff cells co-cultured with different groups of Treg cells. Left images are the representative flow cytometric analysis of Teffs in different groups. Right bar diagram shows the proliferation ratio of Teffs. Data were displayed as mean ± SEM; *P < 0.05; ns *P* > 0.05.

Whether the function reversal of CD4^+^ Tregs concerns its change in glucose metabolism cause our curiosity. To investigate this, we used glycolysis inhibitor 2-deoxy-d-Glucose (2-DG) and glycolysis key enzyme inhibitor Galloflavin to inhibit glycolysis of CD4^+^ Tregs in the co-culture microenvironment of OC cells. Then CFSE staining was used to detect the effect of CD4^+^ Tregs on the proliferation of Teff cells. As shown in Fig. 4A–C, the expressions of glucose metabolism-related genes and proteins in CD4^+^ Treg cells all decreased after inhibitor treatment, which indicated that the inhibition was effective.

After co-culturing CD4^+^ Tregs with SKOV3 for 3 days, ssRNA40 was used to activate the TLR8 signaling of CD4^+^ Tregs and the effect of CD4^+^ Tregs on the proliferation of Teffs was detected by proliferation assay. As shown in Fig. 4D, the proliferation level of Teffs in the treatment group with TLR8 ligand ssRNA40 was significantly higher than that in the untreated group (*P* < 0.05), which was consistent with Fig. 3A. Meanwhile, after 2-DG and galloflavin treatment, the inhibition of CD4^+^ Tregs on the proliferation of Teff cells was also weakened (*P* < 0.05). And we used 2-DG and Galloflavin to inhibit the glycolysis of CD4^+^ Tregs, then half of them were treated with ssRNA40 while the other half were not. After above treatments, CD4^+^ Tregs were co-cultured with Teffs. At this time, the suppression ability of CD4^+^ Tregs with TLR8 activation was not significantly different from that of Tregs treated by inhibitors only (Fig. 4D right). These results suggested that activation of TLR8 signaling may reverse the suppressive function of CD4^+^ Tregs, resulting from glucose metabolism changes.

### TLR8 signaling affects mTOR pathway negatively in CD4^+^ Treg cells co-cultured with SKOV3

**Fig. 5:**
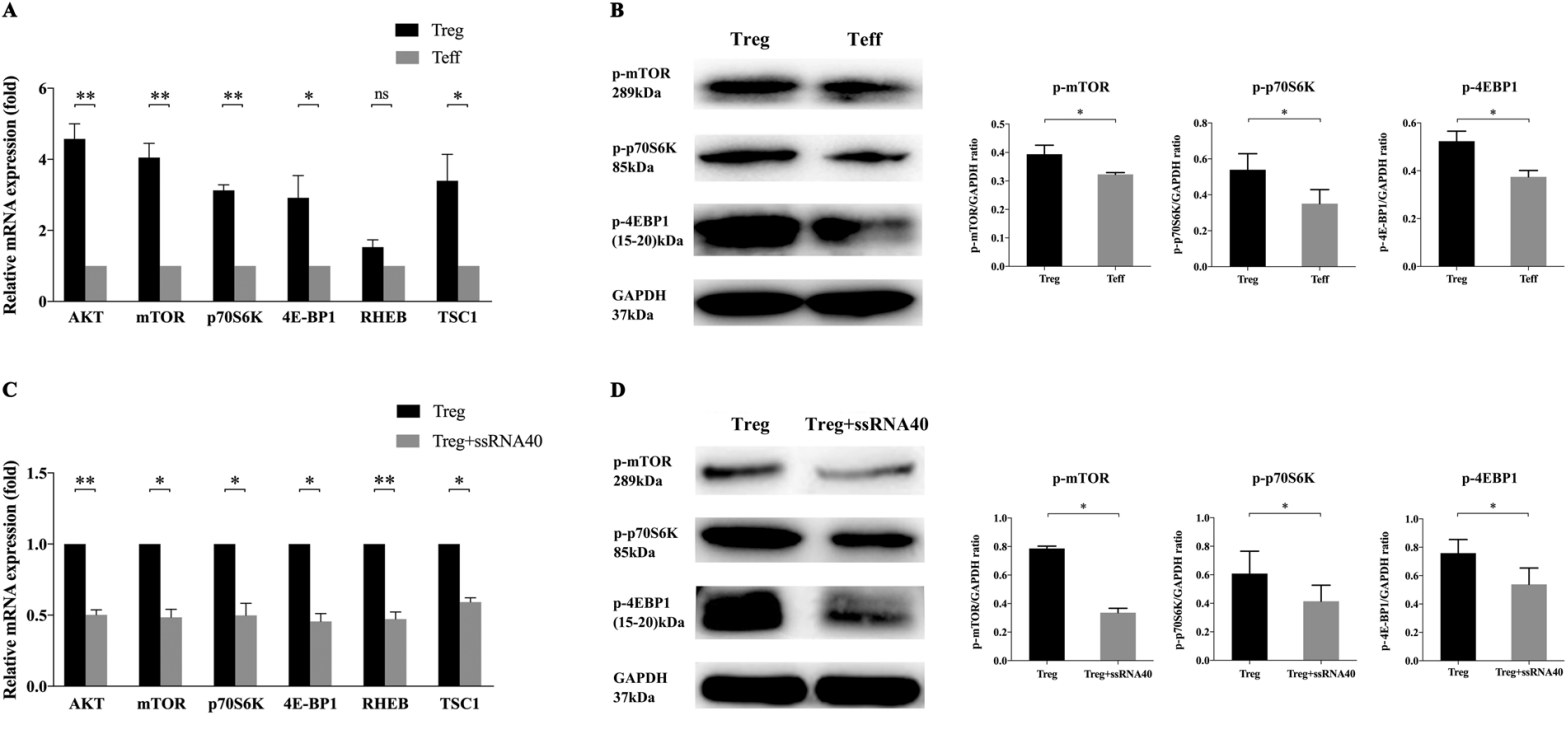
Effects of TLR8 ligands on mTOR pathway in CD4^+^ Tregs co-cultured with SKOV3. **A** Expression levels of key genes in mTOR pathway (AKT, mTOR, p70S6K, 4E-BP1, RHEB, TSC1) in CD4^+^ Tregs with quantitative real-time PCR. Expression levels of each gene were normalized to β-actin expression level and adjusted to the levels in CD4^+^ Teffs (served as 1). Data shown are mean ± SEM; *P < 0.05; **P < 0.01; ns *P* > 0.05. **B** Expression levels of key proteins in mTOR pathway (p-mTOR, p-p70S6K, p-4E-BP1) detected by western blot. The upper three panels show the western blot analysis results. The bottom panel shows the protein expressions analyzed quantitatively and compared with GAPDH expression with a densitometer. Results shown in the histogram are mean ± SEM; *P < 0.05. **C** Expression levels of genes in mTOR pathway (AKT, mTOR, p70S6K, 4E-BP1, RHEB, TSC1) in CD4^+^ Tregs after treated by ssRNA40 with quantitative real-time PCR. Expression levels of each gene were normalized to β-actin expression level and adjusted to the levels in CD4^+^ Tregs without TLR8 activation (served as 1). Data shown are mean ± SEM; *P < 0.05; **P < 0.01. **D** Expression levels of proteins in mTOR pathway (p-mTOR, p-p70S6K, p-4E-BP1) detected by western blot. The upper three panels show the western blot analysis results. The bottom panel shows the protein expressions analyzed quantitatively and compared with GAPDJ expression with a densitometer. Results shown in the histogram are mean ± SEM; *P < 0.05.

Many studies have shown that mammalian target of rapamycin (mTOR) signaling has an important role in T-cell differentiation and function as a glucose metabolism-related pathway^28^. As shown in Fig. 5A and B, the expression levels of mTOR pathway were significantly higher in CD4^+^ Tregs than that of Teffs, suggesting that mTOR pathway is positively correlated with the level of glucose metabolism. Genes tested included mTOR, protein kinase B (AKT), p70 ribosomal protein S6 kinase (p70S6K), e IF4E-binding protein 1 (4E-BP1), ras-homolog enriched in brain (RHEB) and tuberous sclerosis 1 (TSC1).We also detected the key protein levels of mTOR pathway in CD4^+^ Tregs co-cultured with SKOV3 or not and got the same conclusion (Fig. S5). Then we tested the expression levels of mTOR pathway in CD4^+^ Treg cells with or without TLR8 activation. Compared to cells without ssRNA40 treatments, TLR8 signaling downregulated mTOR pathway obviously (Fig. 5C, D). According to the above results, we supposed that TLR8-mediated changes in CD4^+^ Treg metabolism and function may have a potential mechanistic relationship with mTOR signaling.

## Discussion

In addition to the effect of cell state, the metabolic characteristics of different subsets of T cells are also different. In the past, it was generally believed that most of the Teffs were metabolized by glycolysis, whereas Tregs were metabolized by oxidative phosphorylation of glucose and fatty acids^16–18^. However, in recent years, this dichotomy is thought not accurate. Some researchers reported that ex vivo Treg cells were highly glycolytic while Teff cells used predominantly fatty-acid oxidation (FAO). When cultured in vitro, Treg cells engaged both glycolysis and FAO to proliferate, whereas Teff cells mainly relied on glucose metabolism^19,20^. For instance, the metabolism features of thymic derived Tregs (tTregs) with Foxp3 constitutive expression are actually similar to that of Teffs, because they rely on glycolysis pathway for proliferation. On the contrary, for Tregs induced in the peripheral (pTregs) with Foxp3 non-constitutively expressed, oxidative phosphorylation is preferred to promote their proliferation and functional adaptation^7,21,22^. However, in the specific environment of proliferation, the pathway of glucose metabolism of these Tregs can undergo temporary environment-related metabolic transformation and become glycolysis^23^. It has been reported that the transformation of this metabolic process is regulated by a variety of signaling pathways: Akt can directly upregulate the enzymes in the glycolysis pathway or activate mTOR in downstream to enhance glycolysis^24^. Procaccini et al.^25^ found that the discrepancy between different states of Treg cells depended on the elevated activity of the mTOR pathway induced by leptin, which was a dynamic and oscillatory phenomenon necessary for Treg cell expansion to occur. mTOR can enhance the activity of HIF-1α, whereas HIF-1α can upregulate the coding of aerobic glycolysis enzyme at the transcription level. It can also directly regulate Glut1 and LDH-α and interact with PKM2^12^. At the same time, the high expressions of Glut1 and Glut3 are related to the low degree of differentiation in most of the cancer types interviewed, including rectal cancer, breast cancer, lung adenocarcinoma, squamous cell carcinoma, OC, and glioblastoma^26^. De Rosa et al.^27^ also found that the proliferation and function of Treg cells have a tight relationship with glycolysis, which controlled Foxp3 splicing variants containing exon 2 through the glycolytic enzyme Eno1. And the alteration in the suppressive function of Treg cells caused by this is related to human autoimmune diseases. Therefore, we selected eight genes and four proteins related to study the glucose metabolism level of CD4^+^ Tregs and CD4^+^ Teffs.

Our current study found the expression percentages of Glut1 and HIF-1α in CD4^+^ Tregs were significantly higher than that in CD4^+^ Teffs of OC. Besides, the expressions of Glut1 and HIF-1α of CD4^+^ Tregs in peripheral blood of OC were significantly higher than that of BOT and HC. And they also had more active proliferation. It was found that the expression levels of glycolysis related genes and proteins in CD4^+^ Tregs after co-culturing with SKOV3 were significantly increased than those of normal cultured CD4^+^ Tregs and were also higher than those in Teffs. Furthermore, the glucose uptake and glycolysis levels of CD4^+^ Tregs were obviously higher than those of CD4^+^ Teffs too. These results suggested that the glucose metabolism of CD4^+^ Tregs in peripheral blood of OC is not only more active than that of BOT and HC, but also more active than CD4^+^ Teffs. In the environment of OC cells, CD4^+^ Tregs mainly take the way of glycolysis for energy metabolism, and consume more energy than the effector cells, inhibiting the function of the effector cells to promote tumor growth. If we can regulate the immunosuppressive function by inhibiting the glucose metabolism of CD4^+^ Tregs, it will provide a good idea for the immunotherapy of OC.

Many studies have shown that TLR8 can directly inhibit the immunosuppressive function of Tregs. ssRNA40 and ssRNA33, the ligands of TLR8, can reverse the inhibition function of Tregs in the absence of DC. And when TLR8 ligands and its downstream molecules MyD88 are knocked out, the response of Tregs to its ligands disappears, which indicates that the inhibition of TLR8 on Tregs is not achieved by regulating Teffs^28,29^. In addition to direct regulation, TLRs can also indirectly regulate Tregs through antigen-presenting cells (APC) and secretory cytokines. APC activated by TLRs and its secretory cytokines can make Teffs nonresponse state to Tregs^30^. At present, there is no clear conclusion about the mechanism of TLR8 reversing the immunosuppressive function of Tregs. Some studies have shown that TLR8 can reverse the immunosuppressive function of Tregs by regulating the glucose metabolism of Tregs through mTORC1-HIF-1α pathway. TLR8 signaling and Foxp3 can regulate the balance of glucose metabolism of Tregs, thus regulating their proliferation and immunosuppressive function^13,15^.

The current results showed that the after TLR8 signal of CD4^+^ Tregs was activated in the co-culture environment of OC cells, the proliferation ability of naive CD4^+^ T cells was enhanced, indicating that the inhibitory function of Tregs was reversed. At the same time, after activation of TLR8 signal, the glucose uptake and glycolysis levels of CD4^+^ Tregs both decreased, and the expression levels of eight genes and four proteins related to glucose metabolism also decreased. What’s more, TLR8 signaling can downregulation mTOR pathway, which is tightly related to glycolysis metabolism. In addition, the inhibition of CD4^+^ Tregs on the proliferation of Teffs was reversed after 2-DG and Galloflavin were used to inhibit the glycolysis of Tregs. Moreover, using inhibitors first and then activating TLR8 signal did not make the suppressive function of CD4^+^ Tregs significantly different from that inhibiting glycolysis only. These results suggested that TLR8 can affect the glucose metabolism of CD4^+^ Tregs and may reverse the inhibition ability of CD4^+^ Tregs in this way. Go further, the key pathway may be mTOR signaling, which needs to be more investigated.

In summary, OC cells have certain impacts on the glucose metabolism and function of CD4^+^ Tregs, which can be reversed by the regulation of TLR8. Targeting the key metabolizing enzymes and their products of Tregs, regulating the epigenetics of cells through metabolism reprogramming, so as to regulate the immune response of cells to tumor, which provides a new opportunity for immunotherapy of tumor. However, the specific pathway and mechanism of TLR8-mediated regulation of glucose metabolism and reversal of inhibitory function in Tregs are not clear and need further study. In any case, the continuous promotion of the research on metabolism and function regulation of Tregs in OC, as well as the continuous exploration of the influence mechanism on the metabolism of Tregs and tumor development, will open up a new way for immunotherapy of OC.

## Methods

### Patients and specimens

This research was authorized by the Ethical Committee of the First Affiliated Hospital of Nanjing Medical University (Nanjing, China, 2017-SRFA-064), and written inform consent was obtained from all patients. Fresh peripheral blood samples were obtained from OC and BOT patients treated at Nanjing maternity and child health care hospital from 2019 to 2020. None of these patients had received radiotherapy or chemotherapy before specimen collection. HC samples were from healthy donors who did a physical examination at the First Affiliated Hospital of Nanjing Medical University in 2020. The clinical data of the patients were also collected for analysis.

### Separation of mononuclear cells

Peripheral blood mononuclear cells (PBMCs) were obtained from fresh PB using Ficoll-Hypaque (TBD, Tianjin, China) density gradient centrifugation.

### Flow cytometry

Freshly isolated PBMCs (1 × 10^6^) were washed and incubated with fluorophore-conjugated monoclonal antibodies including fluorescein isothiocyanate (FITC)-anti-CD4, APC-anti-CD25, BV421-anti-CD127, Alex Fluor 700-anti-Glut1 (from BD Biosciences, San Jose, CA, USA or R&D, Minnesota, USA) for 20 min at room temperature in the dark. For intracellular staining, PBMCs (1 × 10^6^) were washed, fixed, permeabilized, and stained with PE-anti-HIF-1α (R&D, Minnesota, USA) according to the manufacturer’s protocol. For intranuclear transcription factor detection, cells (1 × 10^6^) were washed, fixed, permeabilized, and stained with PE-anti-Foxp3 (BD Biosciences, San Jose, CA, USA) according to the manufacturer’s protocol. For Ki67 detection, cells (1 × 10^6^) were washed, fixed, permeabilized, and stained with PE-anti-ki67 (BD Biosciences, San Jose, CA, USA) according to the manufacturer’s protocol. Fluorescence data were collected on a FACS Aria II (BD Biosciences, San Jose, CA, USA) and analyzed with FlowJo software (Tree Star, Ashland, OR, US).

### Cell sorting and cell line culture

CD4^+^ Tregs and Teffs were sorted from PBMCs by fluorescence-activated cell sorting (FACS), following staining with FITC-anti-CD4, APC-anti-CD25, PE-anti-CD127 (all from BD Biosciences, San Jose, CA, USA), using a FACS Aria II cell sorter; the purity of the sorted cells was >95%.

The human OC cell line SKOV3 was purchased from ATCC (American Type Culture Collection, Manassas, VA, USA), and cultured in McCoy’s 5 A medium (Invitrogen, Carlsbad, CA, USA) supplemented with 10% FBS at 37 °C in 5% CO_2_.

### Amplification of CD4^+^ Treg and Teff cells in vitro

Sorted CD4^+^ Tregs and Teffs were washed and resuspended in X-VIVO15 medium (LONGZA, Basle, Switzerland) with 5% human AB serum (Gemini, Woodland, CA, USA) and 500 U/ml IL-2 (Peprotech, Rocky Hill, NJ, USA) was added to Tregs suspension. Spread 1 × 10^5^ cells per well in 96-well round-bottom plate and then add 20 μl/well anti-CD3/CD28-coated microbeads (Invitrogen, Carlsbad, CA, USA). Cell suspension was divided into two well equally when cells expanded double. Cells were cultured for 14 days.

### Co-culture experiments

CD4^+^ Tregs and Teffs (1.6 × 10^6^/ml) amplified from healthy PB were cultured with human OC cell line SKOV3 (4 × 10^5^/ml) separated by a transwell chamber with X-VIVO15 medium in 24-well plates at 37 °C in 5% CO_2_. After 3 days, T cells were harvested for following experiments.

### TLR8 signaling activation

Amplified CD4^+^ Tregs and Teffs after co-cultured with SKOV3 were harvested and resuspended in X-VIVO15 medium with 5% human AB serum. Spread 3 × 10^5^ cells per well in 96-well round-bottom plate and add 3 μg/ml TLR8 ligand ssRNA40 (Invivogen, USA). After 2 days, T cells were harvested for the following experiments.

### RNA isolation and quantitative real-time PCR

Total RNA was isolated from CD4^+^ Treg and Teff cells using RNeasy Micro kit (Qiagen, Dusseldorf, Germany), and then reverse transcribed to cDNA with Prime Script RT Master Mix (Takara, Otsu, Japan) according to the manufacturer’s instructions. Genes related to glucose metabolism mRNA levels were quantified with TB Green and a 7500 Real-Time PCR system (Applied Biosystems; Life Technologies, Grand Island, NY, USA). The primer sequences were in Table 1. The relative expression of these target genes normalized by β-actin was calculated as 2^−ΔCt^.

**Table 1:** List of PCR primer sequences.

Gene name	Upstream primers (5′→3′)	Downstream primers (5′→3′)
Glut1	TTGGCTCCGGTATCGTCAAC	GCCAGGACCCACTTCAAAGA
Glut3	GCTCTCTGGGATCAATGCTGTGT	CTTCCTGCCCTTTCCACCAGA
HIF-1α	CCATTAGAAAGCAGTTCCGC	TGGGTAGGAGATGGAGATGC
TPI	AGGCATGTCTTTGGGGAGTC	AGTCCTTCACGTTATCTGCGA
GPI	AGGCTGCTGCCACATAAGGT	AGCGTCGTGAGAGGTCACTTG
LDHα	CCAGCGTAACGTGAACATCTT	CCCATTAGGTAACGGAATCG
ENO1	TCATCAATGGCGGTTCTCA	TTCCCAATAGCAGTCTTCAGC
PKM2	GCCGCCTGGACATTGACTC	CCATGAGAGAAATTCAGCCGAG
mTOR	ATGCTTGGAACCGGACCTG	TCTTGACTCATCTCTCGGAGTT
p70S6K	AGCCAAAGATCACATAGTGGT	GGCAGATATTCAACTTTGTCCA
AKT	CTGAGATTGTGTCAGCCCTGGA	CACAGCCCGAAGTCTGTGATCTTA
4E-BP1	TCACACTCAGACTCCGAGA	GAATGCCCTTCCTTAGCAA
RHEB	TTGTGGACTCCTACGATCCAA	GGCTGTGTCTACAAGTTGAAGAT
TSC1	ATAGCTGTTACCTCGACGAGT	TGCAGGGAGACCTCTATGTCC
β-actin	GAGCTACGAGCTGCCTGACG	GTAGTTTCGTGGATGCCACAG

### Western blotting analysis

CD4^+^ Tregs or Teffs after various treatments were washed and harvested. Whole-cell lysates of the T cells were prepared for western blot analyses. Western blots were developed with Chemiluminescent Substrate (Thermo Fisher Scientific, Waltham, Massachusetts, USA). The rabbit polyclonal antibodies used in western blotting are anti-Glut1, anti-LDHα, anti-PKM2, anti-GPI, anti-Eno1, anti-phostho-mTOR, anti- phostho-p70S6K, anti- phostho-4E-BP1, anti-β-actin and anti-GAPDH (Cell Signaling Technology, Boston, USA).

### Glucose uptake assay

CD4^+^ Tregs or Teffs were washed and resuspended in medium without serum to starve cells overnight. Next morning used glucose uptake analysis kit (Biovision, San Francisco, USA) to detect the glucose uptake levels of cells according to the manufacturer’s protocol.

### Glycolysis level detection

The supernatant of CD4^+^ Tregs or Teffs after various treatment were collected. Glycolysis cell-based assay kit (Cayman, Michigan, USA) was used to detect glycolysis levels. All operations were according to the manufacturer’s protocol.

### Proliferation assay of naive CD4^+^ T cells

CD4^+^ Tregs were cultured with SKOV3 and activated TLR8 signaling or not. Naive CD4^+^ T cells were negatively selected from healthy donors using a naive CD4^+^ T Cell Isolation Kit II (Miltenyi Biotec, Germany). CD4^+^ Tregs (1 × 10^5^) collected from the two groups were co-cultured with naive CD4^+^ T cells at a ratio of 1:1 in round-bottom 96-well plates containing anti-CD3 and anti-CD28 antibody (1 μg/ml; eBioscience, San Diego, CA, USA). PBMCs (5 × 10^4^) irradiated with 40 Gy were added to the culture as APCs. After 56 h of culture, [^3^H] thymidine was added at a final concentration of 1 μCi/well and cultured for an additional 16 h. The incorporation of [^3^H] thymidine was measured with a liquid scintillation counter.

### Proliferation assay of CD4^+^ Teff cells

CD4^+^ Tregs were inhibited glycolysis by ssRNA40, glycolysis inhibitor 2-DG and LDH-α inhibitor Galloflavin. Cells without inhibition as control group. CD4^+^ Tregs (1 × 10^5^) collected from the four groups were co-cultured with CD4^+^ Teffs at a ratio of 1:1 in round-bottom 96-well plates containing anti-CD3 and anti-CD28 antibody (1 μg/ml). CD4^+^ Teffs were stained by CFSE dye (2 μM, Invitrogen, Carlsbad, CA, USA) according to the manufacturer’s protocol. And add CD4^+^ Teffs (1 × 10^5^) to a well without anti-CD3 and anti-CD28 antibody as the parent peak. After 96 h of culture, proliferation levels of Teffs were detected by flow cytometry.

### Statistical analysis

Data were analyzed with SPSS (Statistical Package for the Social Science) 20.0 software (IBM Corp, Armonk, NY, USA) and are expressed as the mean ± standard error (SEM). Differences between the two groups were evaluated using Student’s *t* test or the non-parametric Mann–Whitney *U* test. *P* values < 0.05 were considered statistically significant.

## Supplementary information

Figure S1 Glucose metabolism-related factors expression of CD4^+^ Tregs and Teffs in peripheral blood

Figure S2 Ki67 expression of CD4^+^ Tregs in peripheral blood and SKOV3 co-cultured environment

Figure S3 Establishment of the amplification system of T cells in vitro

Figure S4 Expression levels of glucose metabolism-related genes and proteins in CD4^+^ Teffs after TLR8 activation

Figure S5 Expression levels of mTOR pathway in CD4^+^ Tregs and Teffs in SKOV3 co-cultured environment

Supplementary figure legends

## References

[1] Siegel RL, Miller KD, Jemal A (2020). Cancer statistics, 2020. CA Cancer J. Clin..

[2] Yigit R, Massuger LFAG, Figdor CG, Torensma R (2010). Ovarian cancer creates a suppressive microenvironment to escape immune elimination. Gynecol. Oncol..

[3] Shah MA, Schwartz GK (2001). Cell cycle-mediated drug resistance: an emerging concept in cancer therapy. Clin. Cancer Res..

[4] Zou W (2005). Immunosuppressive networks in the tumour environment and their therapeutic relevance. Nat. Rev. Cancer.

[5] Shevach EM (2009). Mechanisms of foxp3+ T regulatory cell-mediated suppression. Immunity.

[6] Teng MWL, Ritchie DS, Neeson P, Smyth MJ (2011). Biology and clinical observations of regulatory T cells in cancer immunology. Curr. Top. Microbiol. Immunol..

[7] Palmer CS, Ostrowski M, Balderson B, Christian N, Crowe SM (2015). Glucose metabolism regulates T cell activation, differentiation, and functions. Front. Immunol..

[8] Lunt SY, Vander Heiden MG (2011). Aerobic glycolysis: meeting the metabolic requirements of cell proliferation. Annu. Rev. Cell Dev. Biol..

[9] Bauer DE (2004). Cytokine stimulation of aerobic glycolysis in hematopoietic cells exceeds proliferative demand. FASEB J..

[10] Chang C-H (2015). Metabolic competition in the tumor microenvironment is a driver of cancer progression. Cell.

[11] Hu Z, Zou Q, Su B (2018). Regulation of T cell immunity by cellular metabolism. Front. Med..

[12] Schenten D (2014). Signaling through the adaptor molecule MyD88 in CD4+ T cells is required to overcome suppression by regulatory T cells. Immunity.

[13] Gerriets VA (2016). Foxp3 and Toll-like receptor signaling balance T cell anabolic metabolism for suppression. Nat. Immunol..

[14] Walker LSK (2009). Regulatory T cells overturned: the effectors fight back. Immunology.

[15] Li L (2019). TLR8-mediated metabolic control of human treg function: a mechanistic target for cancer immunotherapy. Cell Metab..

[16] Coloff JL (2011). Akt requires glucose metabolism to suppress Puma expression and prevent apoptosis of leukemic T cells. J. Biol. Chem..

[17] Michalek RD (2011). Cutting edge: distinct glycolytic and lipid oxidative metabolic programs are essential for effector and regulatory CD4+ T cell subsets. J. Immunol..

[18] Shan J, Jin H, Xu YT, Cell (2020). Metabolism: a new perspective on Th17/Treg cell imbalance in systemic lupus erythematosus. Front. Immunol..

[19] Li L (2019). TLR8-mediated metabolic control of human treg function: a mechanistic target for cancer article TLR8-mediated metabolic control of human Treg function: a mechanistic target for cancer immunotherapy. Cell Metab..

[20] Procaccini C (2016). The proteomic landscape of human ex vivo regulatory and conventional t cells reveals specific metabolic requirements. Immunity.

[21] Alon R (2017). A sweet solution: glycolysis-dependent Treg cell migration. Immunity.

[22] Tanimine N (2019). Differential effects of 2-deoxy-D-glucose on in vitro expanded human regulatory T cell subsets. PLoS ONE.

[23] Pacella I, Piconese S (2019). Immunometabolic checkpoints of treg dynamics: adaptation to microenvironmental opportunities and challenges. Front. Immunol..

[24] Lv L (2011). Acetylation targets the M2 isoform of pyruvate kinase for degradation through chaperone-mediated autophagy and promotes tumor growth. Mol. Cell.

[25] Procaccini C (2010). An oscillatory switch in mTOR kinase activity sets regulatory T cell responsiveness. Immunity.

[26] Cluxton D, Petrasca A, Moran B, Fletcher JM (2019). Differential regulation of human treg and Th17 cells by fatty acid synthesis and glycolysis. Front. Immunol..

[27] De Rosa V (2015). Glycolysis controls the induction of human regulatory T cells by modulating the expression of FOXP3 exon 2 splicing variants. Nat. Immunol..

[28] Peng G (2005). Immunology: Toll-like receptor 8-mediated reversal of CD4+ regulatory T cell function. Science.

[29] Voo KS (2014). Targeting of Toll-like receptors inhibits CD4+ regulatory T cell function and activates lymphocytes in human PBMCs. J. Immunol..

[30] Oberg HH, Juricke M, Kabelitz D, Wesch D (2011). Regulation of T cell activation by TLR ligands. Eur. J. Cell Biol..

