# Supplementary figures and images for: Glucose metabolism characteristics and TLR8-mediated metabolic control of CD4+ Treg cells in ovarian cancer cells microenvironment
Source: Cell Death Dis. 2021 Jan 7;12(1):22. doi: 10.1038/s41419-020-03272-5 (PMC7790820; doi:10.1038/s41419-020-03272-5)

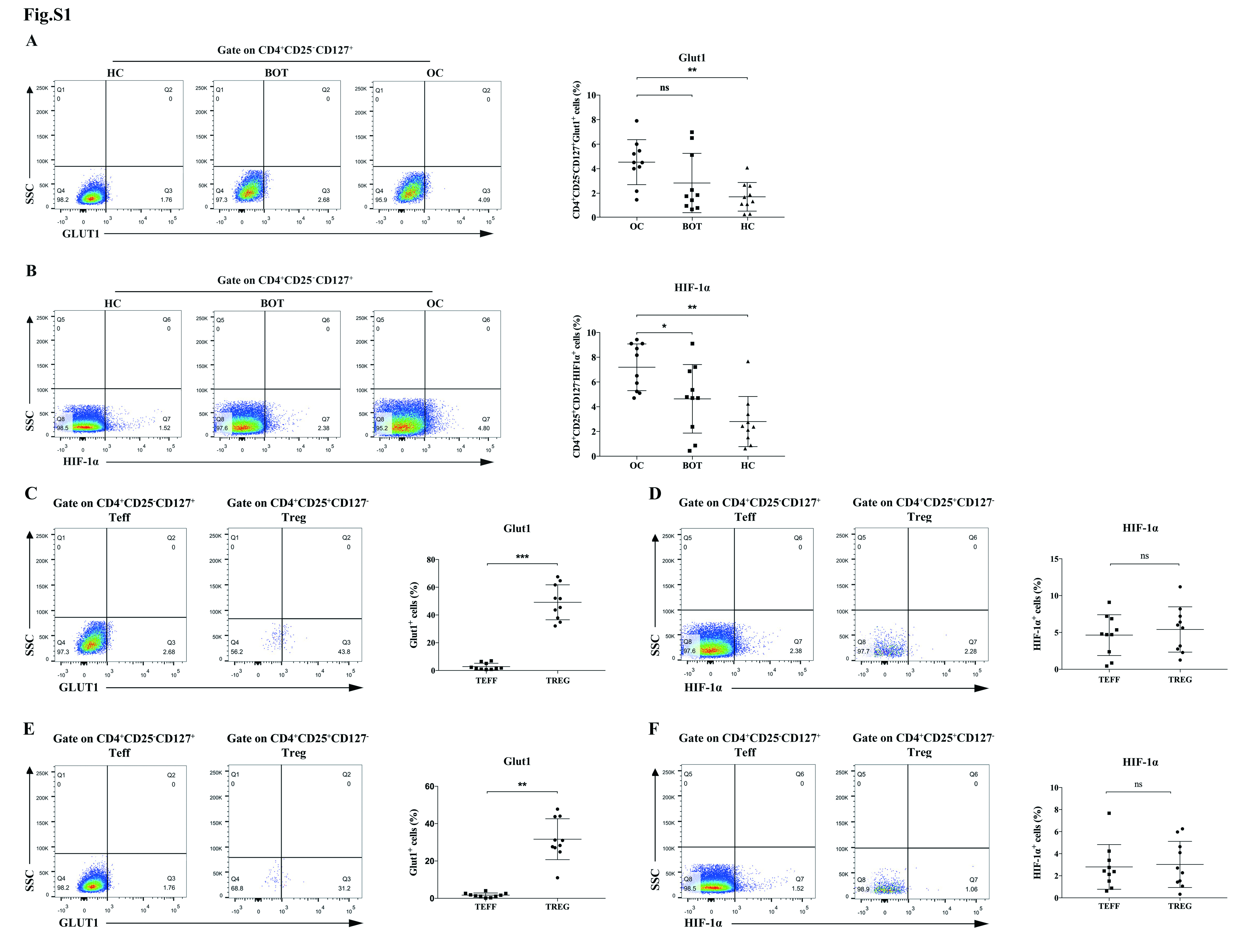

Supplement: Supplementary file 1 — Figure S1 Glucose metabolism-related factors expression of CD4+ Tregs and Teffs in peripheral blood [file 41419_2020_3272_MOESM1_ESM.jpg]

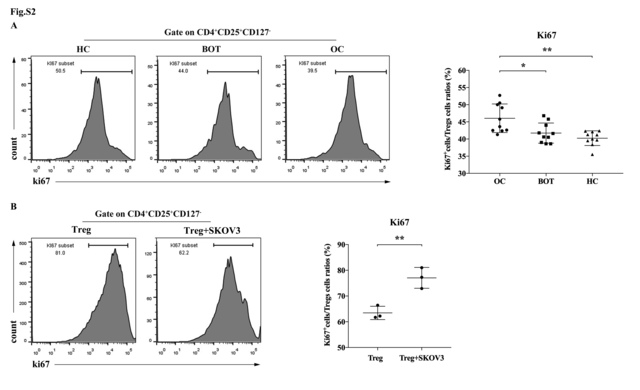

Supplement: Supplementary file 2 — Figure S2 Ki67 expression of CD4+ Tregs in peripheral blood and SKOV3 co-cultured environment [file 41419_2020_3272_MOESM2_ESM.jpg]

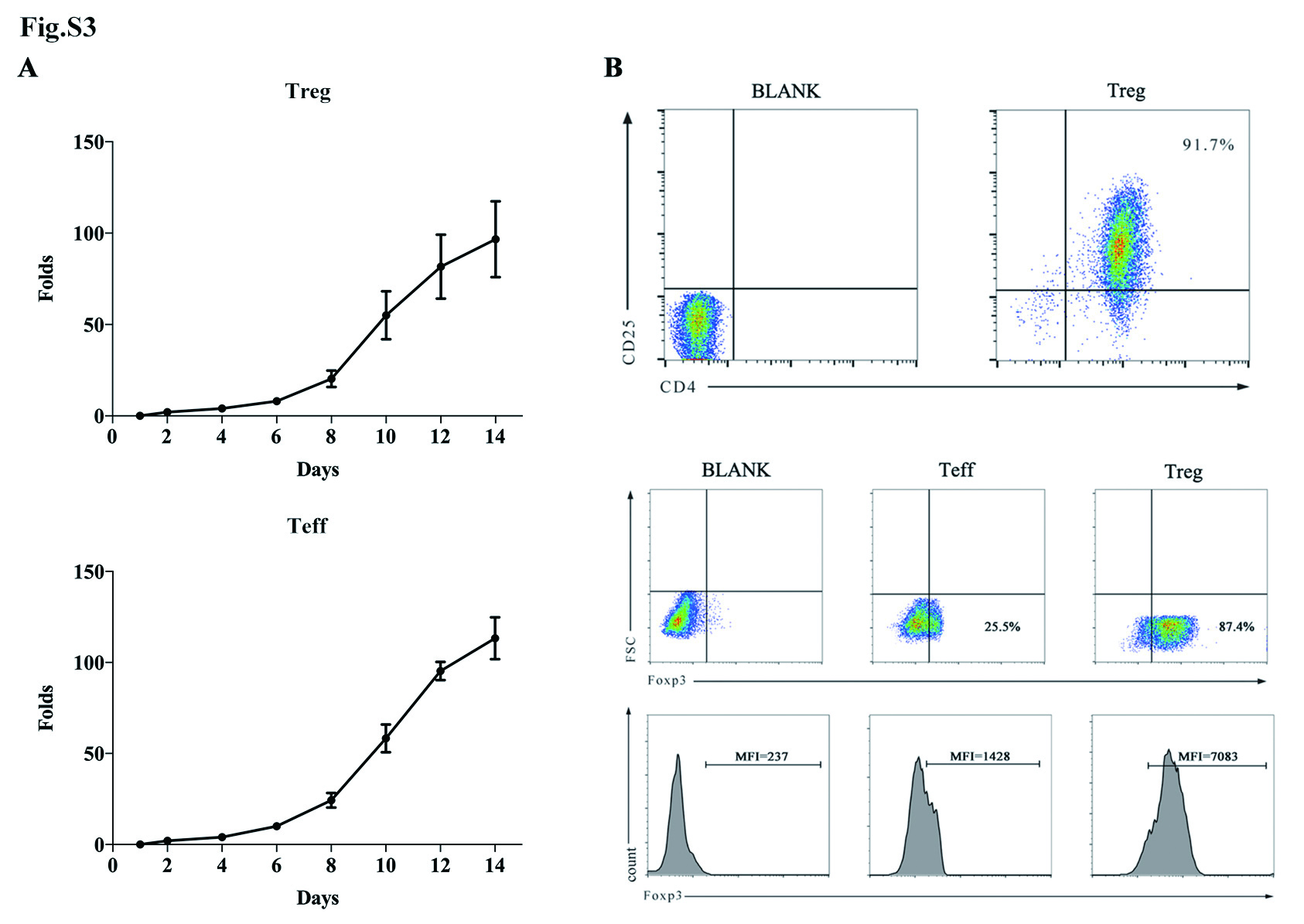

Supplement: Supplementary file 3 — Figure S3 Establishment of the amplification system of T cells in vitro [file 41419_2020_3272_MOESM3_ESM.jpg]

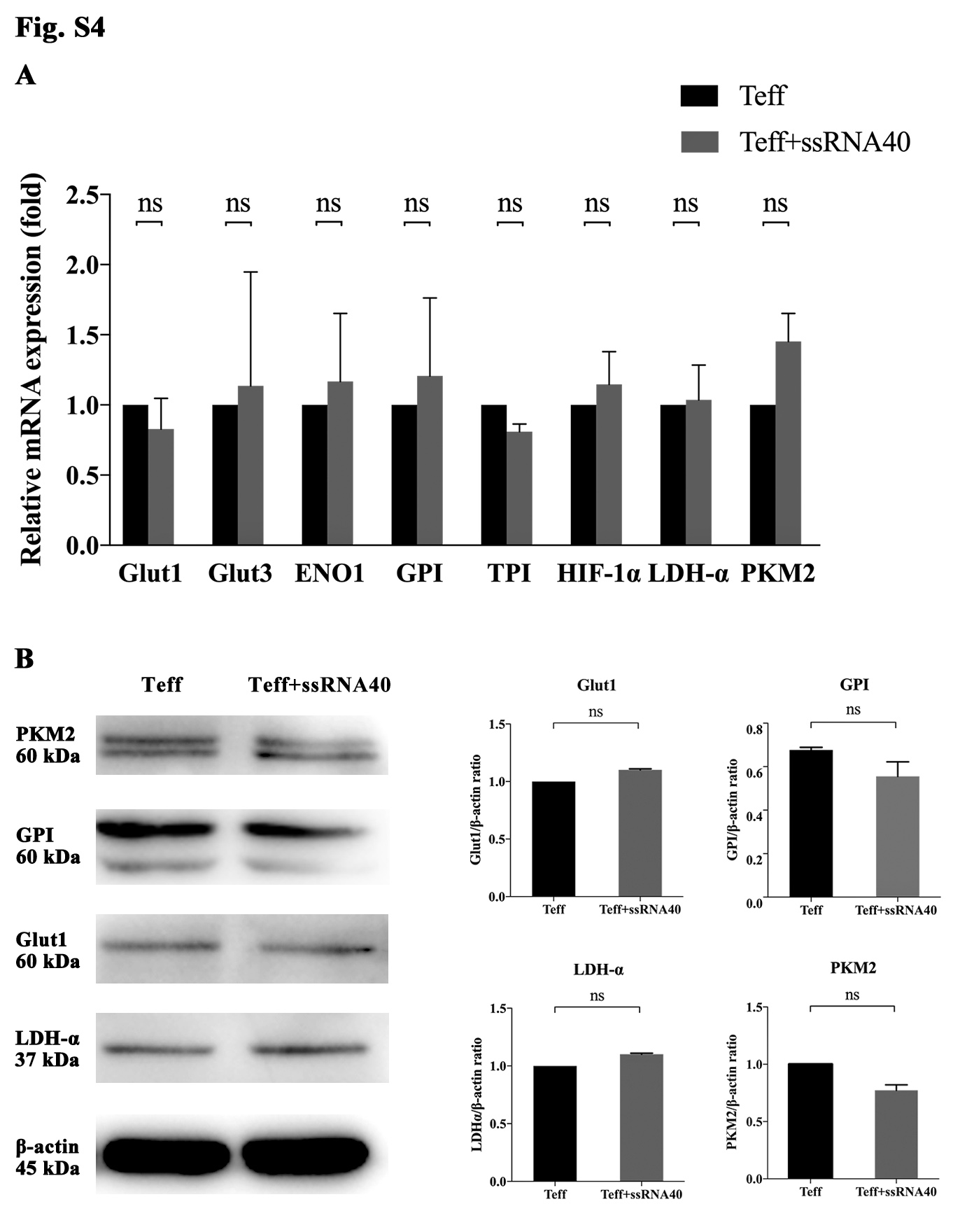

Supplement: Supplementary file 4 — Figure S4 Expression levels of glucose metabolism-related genes and proteins in CD4+ Teffs after TLR8 activation [file 41419_2020_3272_MOESM4_ESM.jpg]

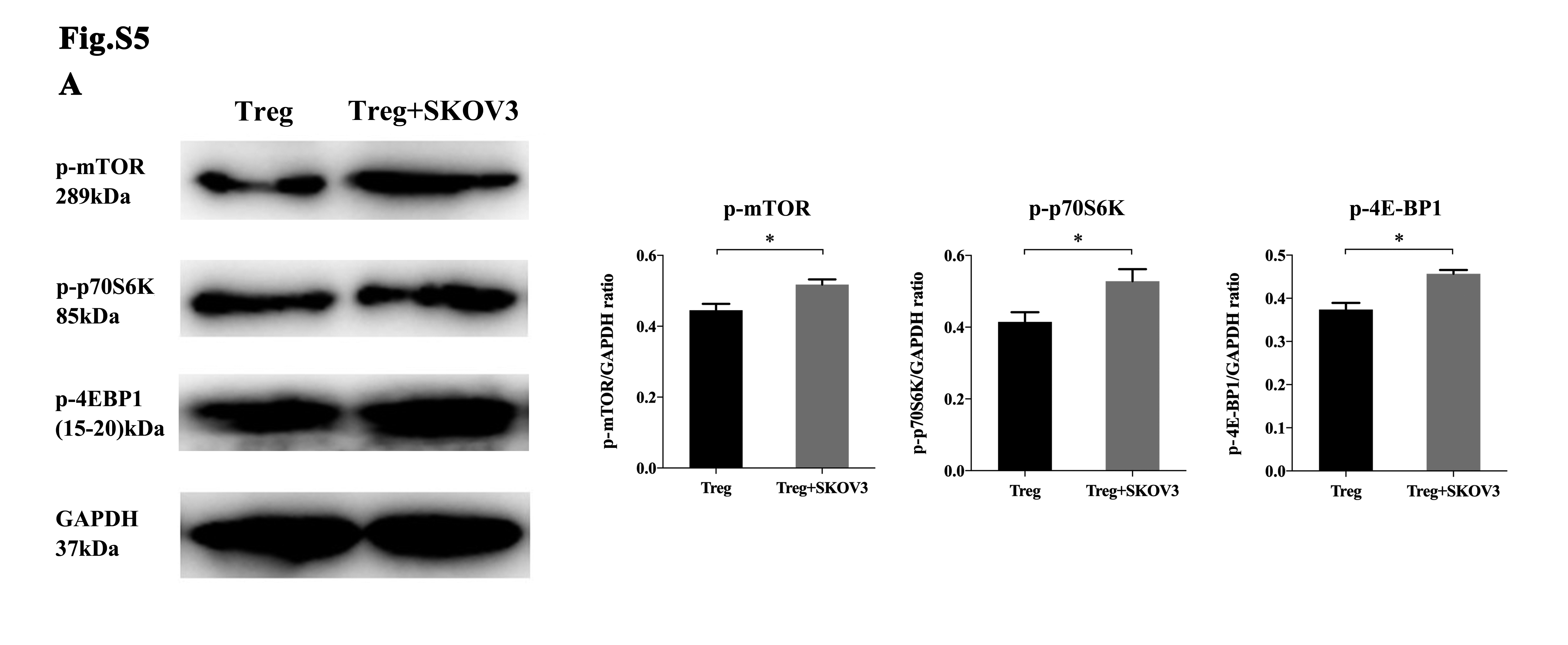

Supplement: Supplementary file 5 — Figure S5 Expression levels of mTOR pathway in CD4+ Tregs and Teffs in SKOV3 co-cultured environment [file 41419_2020_3272_MOESM5_ESM.jpg]
