# Supplementary material for: Glucose metabolism characteristics and TLR8-mediated metabolic control of CD4+ Treg cells in ovarian cancer cells microenvironment
Source: Cell Death Dis. 2021 Jan 7;12(1):22. doi: 10.1038/s41419-020-03272-5 (PMC7790820; doi:10.1038/s41419-020-03272-5)
Supplement: Supplementary file 6 — Supplementary figure legends [file 41419_2020_3272_MOESM6_ESM.docx]

**Supplementary figure legend**

**Figure S1 Glucose metabolism related factors expression of CD4^+^ Tregs and Teffs in peripheral blood**

(A, B) Expression of Glut1 and HIF-1α in CD4^+^ Teffs of human ovarian cancer (n=10) peripheral blood, benign ovarian cancer (n=10) peripheral blood and healthy control (n=10) peripheral blood, as detected via flow cytometry. Left images are the representative flow cytometric analysis and plots are gate on CD4^+^CD25^-^CD127^+^. Right bar diagram summarizes the expression ratios as the mean ± SEM; **P* < 0.05; ***P* < 0.01; ns *P* > 0.05.

(C, D) Left images are the representative flow cytometric analysis of Glut1 and HIF-1α in CD4^+^ Tregs and Teffs of BOT (n=10) and HC (n=10) and plots are gate on CD4^+^CD25^+^CD127^-^ or CD4^+^CD25^-^CD127^+^. Right bar diagram shows the proportions of Glut1 and HIF-1α in CD4^+^ Tregs and Teffs. Data are displayed as mean ± SEM; ***P* < 0.01; ****P* < 0.001; ns *P* > 0.05.

**Figure S2** **Ki67 expression of CD4^+^ Tregs in peripheral blood and SKOV3 co-cultured environment**

(A) Expression of Ki67 in CD4^+^ Tregs of human ovarian cancer (n=10) peripheral blood, benign ovarian cancer (n=10) peripheral blood and healthy control (n=10) peripheral blood, as detected via flow cytometry. Left images are the representative flow cytometric analysis and graphs are gate on CD4^+^CD25^+^CD127^-^. Right scatter plot diagram summarizes the expression ratios as the mean ± SEM; * *P* < 0.05; ***P* < 0.01.

(B) Expression of Ki67 in CD4^+^ Tregs of healthy donor’s peripheral blood and CD4^+^ Tregs co-cultured with SKOV3, as detected via flow cytometry. Left images are the representative flow cytometric analysis and graphs are gate on CD4^+^CD25^+^CD127^-^. Right scatter plot diagram summarizes the expression ratios as the mean ± SEM; ***P* < 0.01.

**Figure S3 Establishment of the amplification system of T cells in vitro**

(A) The growth curve of CD4^+^ Tregs and Teffs isolated from healthy donor’s peripheral blood.

(B) The upper images are representative flow cytometric analysis and plots are gate on CD4^+^CD25^+^.The mid and bottom images are plots and histograms of CD4^+^ Tregs and Teffs on gate of CD4^+^CD25^+^Foxp3^+^.

**Figure S4 Expression levels of glucose metabolism related genes and proteins in CD4^+^ Teffs after TLR8 activation**

(A) Expression levels of genes related to glucose metabolism (Glut1, Glut3, GPI, TPI, HIF-1α, LDH-α, PKM2, ENO1) in CD4^+^ Teffs after treated by ssRNA40 with quantitative real-time PCR. Expression levels of each gene were normalized to β-actin expression level and adjusted to the levels in CD4^+^ Teffs without TLR8 activation (served as 1). Data shown are mean ± SEM; ns *P* > 0.05.

(C) Expression levels of proteins related to glucose metabolism (Glut1, GPI, LDH-α, PKM2) detected by western blot. The upper four panels show the western blot analysis results. The bottom panel shows the protein expressions analyzed quantitatively and compared with β-actin expression with a densitometer. Results shown in the histogram are mean ± SEM; ns *P* > 0.05.

**Figure S5 Expression levels of mTOR pathway in CD4^+^ Tregs and Teffs in SKOV3 co-cultured environment**

(A) Expression levels of key proteins in mTOR pathway (p-mTOR, p-p70S6K, p-4EBP1) detected by western blot. The upper three panels show the western blot analysis results. The bottom panel shows the protein expressions analyzed quantitatively and compared with GAPDH expression with a densitometer. Results shown in the histogram are mean ± SEM; **P* < 0.05.
